# 

**Published:** 1892-06

**Authors:** 


CONTENTS

Vol. 39. No. 6.

| | PAGE. |
|---|---|
| Talks with Dr. Mandeville | 121 |
| Emetics | 124 |
| British Railway Coaches | 125 |
| Lumbago | 126 |
| The Sunday Question | 127 |
| The Inquisitive Antelope | 128 |
| Their Barefoot Teacher | 129 |
| Offensive Breath | 129 |
| Bashful Bridegrooms | 130 |
| Care of the Teeth | 131 |
| Kissing and the Bible | 132 |
| Treatment of Varicose Veins | 132 |
| Art Must be Free | 133 |
| An Interesting Adventure | 134 |
| Infectious Diseases | 134 |
| Do Animals Talk ? | 135 |
| Girls, Don't Marry in a Hurry | 136 |
| Muscæ Volitantes | 136 |
| Frequency of Thunder Storms | 137 |
| The Keeley Craze | 137 |
| A Remarkable Calculation | 138 |
| Housework as an Exercise | 138 |
| German and English Literature | 139 |
| A Remarkable Coincidence | 139 |
| A Traveling Scholarship | 140 |
| Miscellaneous | 140 |
| Literary | 144 |
| The Poor Man's Gems | 144 |

INDEX TO ADVERTISEMENTS OF HALF A PAGE AND OVER.

| | PAGE. |
|---|---|
| Christian Life | I |
| Hall's Journal | II |
| Bovinine | IV |
| Ayer's Pills | V |
| Hall's Journal Premiums | VII |
| Washington Improvement Co. | VIII |
| Herba Vita | X |
| Royal Baking Powder | XI |
| Buffalo Lithia Water | XI |

